# Supplementary material for: Computer code comprehension shares neural resources with formal logical inference in the fronto-parietal network
Source: eLife. 2020 Dec 15;9:e59340. doi: 10.7554/eLife.59340 (PMC7738180; doi:10.7554/eLife.59340)
Supplement: Supplementary file 2. [file elife-59340-supp2.docx]

Table 2. Activated clusters in each contrast

|  |  | peak MNI coordinates | | | Cluster size | | peak-p  (FWER) | |
| --- | --- | --- | --- | --- | --- | --- | --- | --- |
|  |  | X | Y | Z | vertices | mm2 |  |  |
| Real code > Fake code | |  |  |  |  |  |  | |
| Left hemisphere | |  |  |  |  |  |  | |
| Lateral prefrontal cortex (inferior/middle frontal gyri, precentral and superior frontal sulci) | | -51.7 | 16.8 | 21.1 | 1484 | 2962.45 | 0.0014 | |
| Intra-parietal sulcus/supramarginal and angular gyri | | -45.4 | -54.1 | 39.9 | 846 | 1206.53 | 0.0084 | |
| Middle/inferior temporal gyri and superior temporal sulcus | | -61.4 | -52.7 | -0.6 | 481 | 866.16 | 0.0128 | |
| Calcarine sulcus/lingual gyrus | | -5.3 | -75.4 | 0.1 | 342 | 773.52 | 0.0150 | |
| Right hemisphere | |  |  |  |  |  |  | |
| Medial occipital cortex  Lingual gyrus/pericalcarine sulcus | | 13.2 | -89.9 | -0.4 | 904 | 2313.45 | 0.0036 | |
| Angular gyrus | | 43.3 | -61.7 | 45.4 | 405 | 591.32 | 0.0356 | |
| Inferior frontal sulcus  Peak: middle frontal gyrus | | 47.1 | 24.8 | 30.5 | 274 | 485.05 | 0.0448 | |
| Language > Math | |  |  |  |  |  |  |  |
| Left hemisphere | |  |  |  |  |  |  |  |
| Superior temporal cortex | | -51.6 | -7.7 | -14.0 | 2854 | 5384.96 | 0.0002 | |
| Medial occipital cortex  Peak: lingual gyrus | | -7.4 | -93.7 | -8.7 | 1606 | 3693.39 | 0.0002 | |
| Pars triangularis | | -45.2 | 33.4 | -5.2 | 523 | 1070.54 | 0.0088 | |
| Precuneus | | -4.7 | -49.5 | 27.8 | 445 | 811.74 | 0.0172 | |
| Superior frontal gyrus | | -10.7 | 52.1 | 34.9 | 333 | 706.60 | 0.0224 | |
| Right hemishpere | |  |  |  |  |  |  |  |
| Occiptial pole | | 12.8 | -92.0 | -7.1 | 932 | 2409.48 | 0.0006 | |
| Anterior superior temporal sulcus | | 57.6 | -8.4 | -7.3 | 446 | 1073.59 | 0.0104 | |
| Temporal pole | | 42.5 | 15.9 | -28.1 | 319 | 671.89 | 0.0222 | |
| Posterior superior temporal sulcus | | 51.5 | -34.1 | 4.1 | 298 | 613.10 | 0.027 | |
| Medial orbitofrontal sulcus | | 8.5 | 46.7 | -13.7 | 199 | 547.23 | 0.0336 | |
| Precuneus | | 4.6 | -46.9 | 24.8 | 284 | 502.91 | 0.0376 | |
| Math > Language | |  |  |  |  |  |  |  |
| Left hemisphere | |  |  |  |  |  |  |  |
| Intraparietal sulcus  Peak: supramarginal gyrus | | -50.4 | -38.9 | 44.0 | 1842 | 2797.80 | 0.0004 | |
| Superior frontal sulcus | | -27.2 | 3.5 | 49.7 | 437 | 827.32 | 0.015 | |
| Anterior middle frontal gyrus  Peak: inferior frontal sulcus | | -34.8 | 37.0 | 9.9 | 375 | 702.91 | 0.0204 | |
| Superior insula sulcus | | -28.5 | 20.4 | 8.0 | 283 | 575.54 | 0.0308 | |
| Inferior temporal gyrus | | -53.2 | -59.3 | -9.3 | 291 | 484.12 | 0.0408 | |
| Right hemisphere | |  |  |  |  |  |  |  |
| Intraparietal sulcus | | 31.1 | -46.8 | 43.2 | 2502 | 3491.09 | 0.0002 | |
| Anterior middle frontal gyrus | | 34.7 | 44.9 | 21.4 | 731 | 1406.92 | 0.0058 | |
| Inferior temporal gyrus | | 45.5 | -60.4 | -8.2 | 591 | 1176.76 | 0.0088 | |
| Middle anterior cingulate cortex  Peaks at the middle section of the pericallosal sulcus | | 5.4 | -1.6 | 30.2 | 502 | 1040.37 | 0.0092 | |
| Superior frontal gyrus | | 21.1 | -4.0 | 59.1 | 504 | 934.90 | 0.011 | |
| Anterior insula | | 35.5 | 16.8 | -1.2 | 351 | 688.23 | 0.0218 | |
| Inferior precentral sulcus | | 48.4 | 5.8 | 27.7 | 257 | 549.18 | 0.0348 | |
| Logic > Language | |  |  |  |  |  |  |  |
| Left hemisphere | |  |  |  |  |  |  |  |
| Intraparietal sulcus  Extends medially to precuneus and ventrally to inferior temporal gyrus | | -40.6 | -50.6 | 35.5 | 3347 | 5731.62 | 0.0004 | |
| Pars orbitalis  Extends dorsally to precentral and middle frontal gyri | | -40.9 | 48.2 | -6.8 | 2120 | 4350.10 | 0.002 | |
| Superior frontal gyrus | | -9.3 | 33.4 | 37.2 | 310 | 700.63 | 0.0334 | |
| Pericallosal sulcus | | -7.4 | -32.0 | 30.0 | 333 | 640.21 | 0.0372 | |
| Right hemisphere | |  |  |  |  |  |  |  |
| Intraparietal sulcus Extends medially to precuneus and ventrally to inferior temporal gyrus | | 31.0 | -47.0 | 40.2 | 3699 | 6446.9 | 0.0004 | |
| Middle frontal gyrus Extends to superior frontal sulcus, precentral sulcus, and frontal pole | | 45 | 34.5 | 26.1 | 1908 | 3816.88 | 0.0036 | |
| Pericallosal sulcus | | 8 | -36.3 | 29.1 | 343 | 683.83 | 0.0286 | |
| MSIT | |  |  |  |  |  |  |  |
| Left hemisphere | |  |  |  |  |  |  |  |
| Fusiform gyrus  Extends to intraparietal sulcus | | -30.7 | -67.8 | -10.3 | 4059 | 7701.89 | 0.0002 | |
| Superior precentral sulcus | | -22.1 | -0.9 | 47.0 | 421 | 891.27 | 0.0232 | |
| Superior insula sulcus | | -33.8 | 23.1 | 11.1 | 339 | 690.26 | 0.0324 | |
| Inferior precentral sulcus | | -49 | -2.5 | 37.8 | 314 | 605.36 | 0.0406 | |
| Right hemisphere | |  |  |  |  |  |  |  |
| Intraparietal sulcus Extends to fusiform gyrus | | 40.9 | -40.5 | 35.8 | 3727 | 6865.08 | 0.0002 | |
| Superior insula sulcus | | 29.7 | 26.8 | 4.3 | 438 | 838.39 | 0.0198 | |
| Superior precentral sulcus | | 29.6 | -13.0 | 52.1 | 391 | 748.22 | 0.0232 | |
| Middle anterior cingulate cortex Peak at medial superior frontal gyrus | | 10.5 | 12.0 | 47.7 | 313 | 598.73 | 0.0294 | |
| Inferior precentral sulcus | | 43.1 | 2.4 | 29.8 | 231 | 484.89 | 0.043 | |

Table S3. FDR-corrected p-values for the post-hoc paired t-tests among the overlap between code contrast and the localizer contrasts

| Contrasts  (Left hemisphere) | Language > math | Math > language | Logic > language |
| --- | --- | --- | --- |
| Math > language | 0.607 | -- | -- |
| Logic > language | 0.033 | 0.037 | -- |
| MSIT | 0.624 | 0.210 | 0.001 |

| Contrasts  (Right hemisphere) | Language > math | Math > language | Logic > language |
| --- | --- | --- | --- |
| Math > language | 0.895 | -- | -- |
| Logic > language | 0.103 | < 0.001 | -- |
| MSIT | 0.895 | 0.895 | 0.025 |
