# Supplementary material for: Computer code comprehension shares neural resources with formal logical inference in the fronto-parietal network
Source: eLife. 2020 Dec 15;9:e59340. doi: 10.7554/eLife.59340 (PMC7738180; doi:10.7554/eLife.59340)
Supplement: Supplementary file 3. [file elife-59340-supp3.docx]

Table 3. FDR-corrected p-values for the post-hoc paired t-tests among the overlap between code contrast and the localizer contrasts

| Contrasts  (Left hemisphere) | Language > math | Math > language | Logic > language |
| --- | --- | --- | --- |
| Math > language | 0.607 | -- | -- |
| Logic > language | 0.033 | 0.037 | -- |
| MSIT | 0.624 | 0.210 | 0.001 |

| Contrasts  (Right hemisphere) | Language > math | Math > language | Logic > language |
| --- | --- | --- | --- |
| Math > language | 0.895 | -- | -- |
| Logic > language | 0.103 | < 0.001 | -- |
| MSIT | 0.895 | 0.895 | 0.025 |
